# On vital aid: the why, what and how of validation

**DOI:** 10.1107/S090744490900081X

**Published:** 2009-01-20

**Authors:** Gerard J. Kleywegt

**Affiliations:** a Department of Cell and Molecular Biology, Uppsala University, Biomedical Centre, Box 596, SE-751 24 Uppsala, Sweden

**Keywords:** validation

## Abstract

Limitations to the data and subjectivity in the structure-determination process may cause errors in macromolecular crystal structures. Appropriate validation techniques may be used to reveal problems in structures, ideally before they are analysed, published or deposited. Additionally, such tech­niques may be used *a posteriori* to assess the (relative) merits of a model by potential users. Weak validation methods and statistics assess how well a model reproduces the information that was used in its construction (*i.e.* experimental data and prior knowledge). Strong methods and statistics, on the other hand, test how well a model predicts data or information that were not used in the structure-determination process. These may be data that were excluded from the process on purpose, general knowledge about macromolecular structure, information about the biological role and biochemical activity of the molecule under study or its mutants or complexes and predictions that are based on the model and that can be tested experimentally.

## Abstract 2

The need for validation of macromolecular crystal structures is discussed. A general approach to validation is presented, together with examples of its implementation in the special case of macromolecular crystallography.

## 1. The ‘why’ of validation

X-ray crystallography is a truly marvellous technique that enables us to study the structure of organic and inorganic matter, from simple salts to complex molecular machines. When the technique is applied to biologically relevant molecules or complexes, the results often provide unique insight into and understanding of the relationship between structure and function. For this reason, exciting new crystal structures regularly adorn the front cover of popular and prestigious science journals and magazines. Unfortunately, despite all the progress in instrumentation, methodology, software and protocols over the past 50 years, crystallographers do make mistakes. If the resolution of the crystallographic data is high and the crystallographer is skilled and experienced, there will probably be no major errors in the final model that is deposited and described in a paper. If either the resolution is high or the crystallographer is skilled, but not both, then it is still possible that the there will be no major errors. If the resolution is low and the crystallographer is not very experienced, however, the probability of serious errors becomes dangerously high. In the ‘best-case’ scenario, a flawed structure will be of limited interest and any serious errors will merely pollute the structural archive, the Protein Data Bank (PDB; Berman *et al.*, 2000 ▶). In the worst case, serious errors in a high-profile structure may actually obstruct the progress of science for years to come, which appears to have happened in the case of the five grossly incorrect ABC-transporter structures dis­cussed elsewhere in this issue (Chang *et al.*, 2006 ▶; Miller, 2007 ▶; Korkhov & Tate, 2009 ▶; Jeffrey, 2009 ▶).

It is surprising that the gross errors in the five ABC-transporter structures were not detected earlier in the laboratory in which they were solved. However, the fact that other crystallographers did not seriously challenge the structures either is more easily explained. For the first structure only C^α^ co­ordinates were deposited and for only one of the five structures were the experimental data deposited. The lax attitude of many journals when it comes to (enforcing) their own requirements for deposition of not only models but also ex­perimental data has been criticized for a long time (Jones *et al.*, 1996 ▶; Jones & Kleywegt, 2007 ▶; for a discussion of the history of deposition requirements, see Kleywegt *et al.*, 2004 ▶). Fortunately, this is no longer an issue since the worldwide PDB (wwPDB) has made the deposition of experimental X-ray and NMR data mandatory as of 1 February 2008. This does not mean that errors will no longer be made in the future, but many will hopefully be caught at deposition time and others may be detected at a later stage as the availability of model and data allows the calculation of maps [*e.g.* made available through the Uppsala Electron-Density Server (EDS); Kleywegt *et al.*, 2004 ▶] and the re-refinement of models (Joosten *et al.*, 2009 ▶).

There are two aspects to biomacromolecular X-ray crystallography that invite errors to be made: limitations to the data and subjectivity in the structure-determination process. Diffraction data collected from biological samples is almost invariably weak and noisy. The resolution of the data (and hence its information content) is also usually limited and sometimes it is not possible to collect complete data sets. The data that are collected are always averaged in both space (different molecules inside a crystal, subject to possible static disorder and twinning phenomena) and time (during data collection, with possible deleterious effects of radiation damage, oxidation of the sample or other chemical reactions). Furthermore, any experimental phases will contain (hopefully small) errors and calculated phases will be subject to model bias. Since there is no magical formula to calculate ‘the’ model from the diffraction data, subjectivity on behalf of the crystallographer also plays an important role (Brändén & Jones, 1990 ▶). Of course, different levels of skill and experience already come into play in the crystallization, data-collection and data-processing stages, but mistakes and errors there usually result in failure to solve the structure rather than in a publication and a model that later both need to be retracted (although there are exceptions). Given the same data, no two crystallographers will ever produce identical final models. Their different biases and skill and experience levels will manifest themselves especially during manual model building but also during model refinement (*e.g.* different ways to parameterize a model and the use of different refinement programs and protocols). Even with atomic resolution data, individual decisions will differ during both model building (with respect to possible water molecules, alternative conformations, *etc*.) and refinement [choice of computer program, atomic displacement parameter (ADP) model, treatment of noncrystallographic symmetry, target values and weights of various restraints *etc*.] (Kleywegt, 2000 ▶).

In the first 50 years of biomacromolecular crystallography, there has probably not been a single error-free structure (in the sense that it cannot be improved upon, now or in the future). This means first of all that crystallographers must do their utmost to find and (if possible) fix the major errors in their model prior to deposition and publication. However, since there is no way of keeping even highly suspicious models out of the public database (or of evicting them), users of structural information should also find out about potentially problematic aspects of any model they intend to use as a molecular-replacement probe, to design mutants or ligands, to produce homology models, to compare with related structures or to simulate. In other words, validation is crucial for both the producers and the consumers of biomacromolecular structures and validation tools should be used both to assess the overall quality of a model and to assess the reliability of particularly interesting aspects (active-site residues, interface residues, ligands, inhibitors, cofactors *etc*.).

It is important to realise that errors in crystal structures come in many different shapes and forms. Fortunately, the most serious ones (*e.g.* mistracing the fold of an entire protein domain) are fairly rare because they are normally those that are most easily detected (provided that the crystallographer uses appropriate tools and protocols and does not ignore warning signs). At the other end of the scale are purely clerical errors that do not change the scattering of the model (*e.g.* labelling chemically indistinguishable side-chain atoms in violation of a convention). Many examples of grossly incorrect protein crystal structures have been discussed in the past (Brändén & Jones, 1990 ▶; Kleywegt, 2000 ▶; Davis *et al.*, 2008 ▶) and the ABC-transporter fiasco shows that this is by no means a phenomenon that only occurred in the ‘dark ages’ of protein crystallography. Even when the overall structure of a protein is essentially correct, models of complexes with small-molecule ligands can still have a wide variety of problems, as demonstrated in many papers (Davis *et al.*, 2003 ▶, 2008 ▶; Kleywegt *et al.*, 2003 ▶; Kleywegt, 2007 ▶, and references therein). These problems include errors in the chemical identity of a ligand, errors in the stereochemistry or conformation, the modelling of a phantom ligand or, conversely, misinterpreting ligand density as a chain of water molecules. Interestingly, crystallo­graphers are ‘creative’ and manage to produce new types of errors with regularity. For example, two triclinic structures published in 2004 had seriously incorrect cell constants owing to an error in the wavelength. The resulting models had reasonable geometry (probably thanks to tight restraints) but the *R* values for one of them were in excess of 0.3, which should have raised a few eyebrows given that the resolution was 1.6 Å. After re-refinement in the correct cell the *R* values dropped by ∼0.1 to a much more acceptable level.

In summary, validation of models is crucial. On one hand, it helps the crystallographer to pinpoint aspects of the model that might be in error and need fixing or improving prior to publication and deposition. Validation thus helps to improve the quality and integrity of the structural archive. On the other hand, validation of deposited models informs potential users about the quality of the model as a whole and of important aspects of it. This enables these users to make informed decisions as to the suitability of a model for their specific purposes.

## 2. The ‘what’ of validation

**Figure 1:**
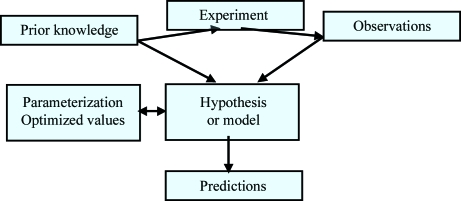
A simple model of hypothesis-driven research in the experimental natural sciences. Prior knowledge leads to a question that can hopefully be answered by carrying out one or more experiments. The observations and the prior knowledge are combined to yield a hypothesis or model, which can be used to make falsifiable predictions. In the case of crystallography, the model consists of the parameterization and the optimal values derived for all parameters.

The dictionary definition of validation alludes to the process of establishing, checking or demonstrating the truth, value or accuracy of, for example, a theory, hypothesis, model or claim. As such, validation is (or rather ought to be) an integral part of every scientific endeavour. It is instructive to consider a simple model of the way in which hypothesis-driven research is carried out in the experimental natural sciences (Fig. 1 ▶). Given an interest in a certain area and a certain amount of prior knowledge, questions can be asked that may be answered through experimentation (*in vivo*, *in vitro* or *in silico*). The results of the experiment (possibly after some data processing) are a set of observations that can be used together with the prior knowledge to construct a model or hypothesis. The hypothesis or model will usually be required to have predictive properties (and thus be falsifiable). This model of doing science is very general: the newly formulated hypothesis or model may pertain to the mechanism of an enzyme reaction, the evolutionary relatedness of a set of species or pro­teins, the feeding or breeding habits of zebrafish as a function of water temperature or, indeed, the three-dimensional structure of a biological macromolecule. In the latter case, the model is a set of point scatterers with assigned atom types (needed to select the appropriate form factors). In most cases, a mapping of the scatterers to specific atoms (based on the chemical diagram of a small molecule or the amino-acid or nucleotide sequence of a biomacromolecule) will also have been made. During refinement, the scatterers are usually considered to be hard spheres connected by springs and a suitable set of parameters is chosen (Cartesian atomic co­ordinates or torsion angles, ADPs, occupancies) and refined subject to a number of restraints and constraints.

In experimental science, errors come in three classes. Random errors lead to noise and affect the precision of measurements. Such errors often have a normal distribution and can be reduced by increasing the number of times a measurement is repeated. Systematic errors, on the other hand, affect the accuracy. These errors introduce a bias in the measurements that arises from, for instance, incomplete knowledge or inadequate experimental design. In contrast to random errors, systematic errors are reproducible and repeating the measurements cannot reduce them. Finally, gross errors or bloopers may occasionally happen, *e.g.* owing to incorrect assumptions, undetected mistakes or instrument malfunction. In favourable cases, gross errors may be detectable as outliers in an experiment. In our simple model of a research project (Fig. 1 ▶), all three kinds of errors can affect the prior knowledge, the experiment and the resulting observations. As a consequence, the model or hypothesis may contain more or less serious errors and these in turn may lead to incorrect predictions. Without validation, there is no way of knowing if the model and the predictions can be trusted at all.

**Figure 2:**
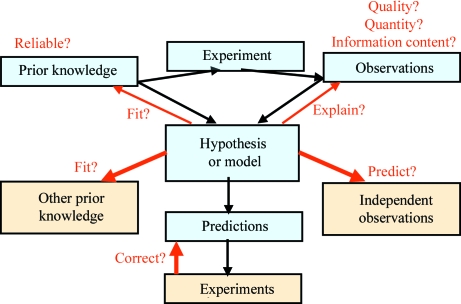
Different ways in which a model or hypothesis can be validated. Firstly, the input to the model itself (prior knowledge and experimental observations) needs to be validated. Secondly, the model or hypothesis should fit in with the prior knowledge and explain the experimental observations (thin arrows). However, the reliability or accuracy of a model is most convincingly demonstrated by its predictive quality with respect to data and information that were not used in the construction of the model (fat arrows). This may entail comparison to new or unused general prior knowledge, to independent observations pertaining to the system under study and to the results of experiments that were designed based on the model.

Our scheme suggests a few obvious ways to validate the model (Fig. 2 ▶). First, the prior knowledge should always be examined critically. As Mark Twain once said: ‘The trouble with most of us is that we know too much that ain’t so’. For instance, any deposited protein structure that is going to be used for molecular replacement, mutant or ligand design, homology modelling or molecular-dynamics simulations ought to be critically examined prior to use. Secondly, the experimental observations should be assessed in terms of quality and quantity. In addition, one should always ascertain that the data have the proper information content to answer the question one is interested in. For instance, a three-dimensional cryo-EM map will typically not be suitable to answer questions about a biological molecule at the level of individual atoms or even residues and a crystal structure (with its crystal contacts) will not in general provide an accurate picture of the dynamics of the molecule in a dilute solution. Thirdly, since the model or hypothesis is based on a synthesis of the experimental observations and the prior knowledge, it is important to check that the model both fits the prior knowledge and explains the observations. These are necessary conditions, but they are not sufficient for validation purposes. The fact that both the prior knowledge and the observations were used directly as input to the model-synthesis process (*e.g.* a crystallographic refinement program) means that such tests only assess whether or not that process was carried out competently. Validation criteria that assess the conformance of model and prior knowledge or of model and experimental data have therefore been termed ‘weak’ (Kleywegt & Jones, 1995*b* ▶).

To validate a model properly, it is necessary to put its pre­dictive power to the test. If the model makes or enables pre­dictions, appropriate experiments can be designed and carried out. If the results are in agreement with the predictions, then the confidence in the model is boosted. It may also be the case that independent observations of the system under study are available (for instance, reflections set aside for cross-validation purposes or data on the effect of certain point mutations on the activity or specificity of an enzyme). If the model explains such observations, despite the fact that they were not used in the construction of the model, then this again increases the confidence in the model. Finally, there may be additional prior knowledge that is not specific to the system under study and that also has not been used in the construction of the model (for instance, the core regions of the Ramachandran plot in which most residues of any newly determined protein crystal structure are expected to cluster). If the model is indeed in agreement with such additional prior knowledge, this can be taken as further evidence that the model is probably reliable. Validation criteria that assess the conformance of a model and any data or information that were not used (directly or indirectly) in the construction of the model have been termed ‘strong’ (or ‘orthogonal’ to the input data and prior knowledge; Kleywegt & Jones, 1995*b* ▶).

## 3. The ‘how’ of validation

The discussion in the previous section, as well as the validation scheme shown in Fig. 2 ▶, was completely general. An obvious follow-up question for a crystallographer is: what makes a crystallographic model a good model? In essence, the answer to this question is simple: a good model is one that makes sense in all possible respects that we can think of, now and in the future (Kleywegt & Jones, 1995*b* ▶).

The model should first of all make sense in terms of chemistry: bond lengths, bond angles and torsion angles should have reasonable values, nonstandard compounds (such as ligands and cofactors) should have correct atom types assigned, stereo-centres should have the correct handedness, planar groups should be flat *etc*. The model should also make sense in terms of physics: there should be no bad contacts or atomic overlaps between atoms that are not covalently bonded (including explicit or implicit H atoms), the core of proteins should be close-packed, there should be lots of favourable hydrogen-bonding interactions, charges should mostly interact with other charges, ADPs should show a reasonable pattern of variation (*e.g.* along a side chain or depending on the degree of solvent exposure) *etc*. Obviously, a model should also make sense in terms of crystallography: the model should explain (and, in the case of unused ‘test-set’ reflections, predict) the experimental data without making unreasonable assumptions and with minimal over-fitting (or ‘under-modelling’), residues should generally fit their own density well *etc*. Many of the conditions of chemistry, physics and crystallography are imposed by the refinement programs, which makes these checks less useful from a validation perspective. The model is expected to adhere to these conditions and therefore good overall values for any such statistics [such as the conventional *R* value and the root-mean-square deviation (r.m.s.d.) of bond lengths from ideal values] do not provide independent proof of the correctness of the model. Indeed, it has been demonstrated that even an insane model (intentionally traced backwards through the electron density) can be refined to yield cosmetically pleasing values of the conventional *R* value and statistics such as the r.m.s. bond-length deviation (Kleywegt & Jones, 1995*a* ▶). On the other hand, any outliers of such quality checks should obviously be examined critically (*e.g.* unusually long or short covalent bonds).

For validation purposes, it is more useful to assess the predictive qualities of a model. In general, three types of data and information can be used for this (Fig. 2 ▶): the fit of the model to general prior knowledge not used in the construction of the model, the prediction of any unused observations specific to the system under study and predictions of properties that can be tested experimentally. The use of a ‘test set’ of reflections for cross-validation purposes (*R*_free_; Kleywegt & Brünger, 1996 ▶) is an important example of the second category. The remainder of these three categories is largely covered by the requirement that a good model should make sense in terms of everything we know about macromolecular structure and in terms of experimental biology and biochemistry. In the case of biomacromolecules, underlying physical and chemical principles manifest themselves in empirically observed structural regularity. For instance, nonbonded interactions are responsible for the limited set of torsion angles that are accessible for the main chain of proteins (as manifested in the appearance of the Ramachandran plot) and for the side chains of amino-acid residues (as manifested in the abundance of preferred rotamer conformations). Interactions between atoms that are separated by many bonds but nearby in space are governed by the same forces, which leads to the favourable interactions observed in high-resolution well refined structures (hydrophobic residues pack together, hydrophilic residues form hydrogen bonds, charged residues in addition may form salt links). Comparison with the structures of related molecules (complexes, mutants, orthologues, paralogues) can also be used for validation purposes: any unexpected differences should be justified or at least receive a plausible explanation. Finally, the whole body of biological and biochemical data of the molecule(s) under study should be examined in light of the structure. Ideally, the structure should explain what is known about the mechanism, substrate preference, the effects of mutations and inhibitors *etc*. If such data are unavailable or deemed unreliable, experiments can be designed based on the model to assess its predictive value.

**Figure 3:**
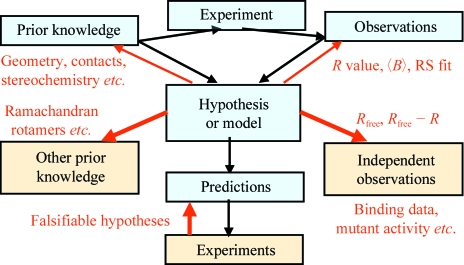
Examples of statistics and checks that can be used to validate biomacromolecular crystal structures, shown in the context of the general approach to validation shown in Fig. 2 ▶.

Fig. 3 ▶ shows some examples of specific validation criteria and quality checks that can be carried out on (protein) crystal structures and how they fit into the general validation scheme that was discussed in the previous section (and shown in Fig. 2 ▶). An extensive set of such criteria is discussed in Kleywegt (2000 ▶). Some of the validation and quality statistics will be calculated by the refinement software, whereas others can be obtained with programs such as *O* (Jones *et al.*, 1991 ▶), *WHATCHECK* (Joosten *et al.*, 2009 ▶) or *MOLPROBITY* (Davis *et al.*, 2007 ▶).

## 4. Concluding remarks: looking back and looking ahead

In 1990, the subject of the CCP4 Study Weekend was ‘Accuracy and Reliability of Macromolecular Crystal Structures’ (Henrick *et al.*, 1990 ▶), a topic that overlaps with that of the 2008 meeting. It was inspired by a number of high-profile cases of serious errors in protein structures (Brändén & Jones, 1990 ▶), including that of the small (S) subunit of tobacco RuBisCO (Schreuder *et al.*, 1990 ▶; Knight *et al.*, 1990 ▶). This structure had essentially been traced backwards through the density in the Los Angeles model (Schreuder *et al.*, 1990 ▶), whereas it was built correctly in the Uppsala model (Knight *et al.*, 1990 ▶). The analysis of the Uppsala model in the proceedings of the 1990 meeting was (one of) the first paper(s) to mention the word ‘validation’ in the title in relation to a macromolecular crystal structure (Knight *et al.*, 1990 ▶). Interestingly, a number of the arguments that Knight and coworkers presented to support the correctness of their model are described as follows:

None of this evidence is dependent on a refined model and instead makes use of known facts about proteins in general and the S subunit of RuBisCO in particular

(Knight *et al.*, 1990 ▶). These arguments included: (i) the heavy-atom-binding sites have chemically plausible ligands, (ii) the S subunit has a well defined hydrophobic core, (ii) conserved residues are found at the S–L subunit interfaces and (iv) a deletion in cyanobacterial S subunits occurs within a loop (Knight *et al.*, 1990 ▶). While today most of these aspects can be investigated on a case-by-case basis using a variety of different programs, there is clearly scope for the development of intelligent tools to perform this automatically and in an integrated fashion.

Validation of macromolecular crystal structures ‘at the gate’ (*i.e.* at the time of deposition at one of the wwPDB nodes) has historically left something to be desired. In an effort to improve this situation, the wwPDB consortium has recently convened a ‘Validation Task Force’ which brings together many experts in the field to advise the wwPDB and also to contribute computer code and programs (Smith *et al.*, 2009 ▶). One goal of this work is to design a brief validation report with the most essential refinement and validation statistics that depositors will receive from the deposition site and that they can send to the journal to which they submit their manuscript. This would help editors and referees to judge the quality of models that have been deposited but are not yet available from the PDB.

Finally, it should be noted that validation demands that the model be based on experimental data (for instance, the Ramachandran plot cannot normally be used to validate homology models). It is conceivable that models that do not satisfy this fundamental *sine qua non* could be (or have been) deposited and published. While some validation techniques will no doubt prove useful for detecting such cases, it is likely that entirely new methods have to be developed to do so reliably. These methods will probably involve assessment of the raw or processed experimental data as well as the fit of the model to the data. Given the gravity of the implications, such methods should have extremely low false-positive rates. However, it is important to realise that validation is something else entirely to fraud detection. Validation can help crystallographers to produce better models (or at least provides a realistic impression of a model’s strengths and shortcomings) and users of structural data to pick the model that best suits their purposes. In short: validation is your friend!

## References

[bb1] Berman, H. M., Westbrook, J., Feng, Z., Gilliland, G., Bhat, T. N., Weissig, H., Shindyalov, I. N. & Bourne, P. E. (2000). *Nucleic Acids Res.* **28**, 235–242. 10.1093/nar/28.1.235 PMC102472 10592235

[bb2] Brändén, C. I. & Jones, T. A. (1990). *Nature (London)*, **343**, 687–689.

[bb3] Chang, G., Roth, C. B., Reyes, C. L., Pornillos, O., Chen, Y. J. & Chen, A. P. (2006). *Science*, **314**, 1875. 10.1126/science.314.5807.1875b 17185584

[bb5] Davis, A. M., St-Gallay, S. A. & Kleywegt, G. J. (2008). *Drug Discov. Today*, **13**, 831–841. 10.1016/j.drudis.2008.06.006 PMC7185550 18617015

[bb4] Davis, A. M., Teague, S. J. & Kleywegt, G. J. (2003). *Angew. Chem. Int. Ed.* **42**, 2718–2736. 10.1002/anie.200200539 12820253

[bb24] Davis, I. W., Leaver-Fay, A., Chen, V. B., Block, J. N., Kapral, G. J., Wang, X., Murray, L. W., Arendall, W. B. III, Snoeyink, J., Richardson, J. S. & Richardson, D. C. (2007). *Nucleic Acids Res.* **35**, W375–W383. 10.1093/nar/gkm216 PMC1933162 17452350

[bb6] Henrick, K., Moss, D. S. & Tickle, I. J. (1990). Editors. *Proceedings of the CCP4 Study Weekend. Accuracy and Reliability of Macromolecular Crystal Structures.* Warrington: Daresbury Laboratory.

[bb7] Jeffrey, P. D. (2009). *Acta Cryst.* D**65**, 193–199. 10.1107/S0907444909001292 PMC2631635 19171975

[bb9] Jones, T. A. & Kleywegt, G. J. (2007). *Science*, **317**, 194–195. 10.1126/science.317.5835.194c 17626864

[bb8] Jones, T. A., Kleywegt, G. J. & Brünger, A. T. (1996). *Nature (London)*, **381**, 18–19. 10.1038/383018a0 8779705

[bb10] Jones, T. A., Zou, J.-Y., Cowan, S. W. & Kjeldgaard, M. (1991). *Acta Cryst.* A**47**, 110–119. 10.1107/s0108767390010224 2025413

[bb23] Joosten, R. P., Womack, T., Vriend, G. & Bricogne, G. (2009). *Acta Cryst.* D**65**, 176–185. 10.1107/S0907444908037591 PMC2631631 19171973

[bb11] Kleywegt, G. J. (2000). *Acta Cryst.* D**56**, 249–265. 10.1107/s0907444999016364 10713511

[bb12] Kleywegt, G. J. (2007). *Acta Cryst.* D**63**, 94–100. 10.1107/S0907444906022657 PMC2483469 17164531

[bb13] Kleywegt, G. J. & Brünger, A. T. (1996). *Structure*, **4**, 897–904. 10.1016/s0969-2126(96)00097-4 8805582

[bb14] Kleywegt, G. J., Harris, M. R., Zou, J., Taylor, T. C., Wählby, A. & Jones, T. A. (2004). *Acta Cryst.* D**60**, 2240–2249. 10.1107/S0907444904013253 15572777

[bb15] Kleywegt, G. J., Henrick, K., Dodson, E. J. & van Aalten, D. M. (2003). *Structure*, **11**, 1051–1059. 10.1016/s0969-2126(03)00186-2 12962624

[bb16] Kleywegt, G. J. & Jones, T. A. (1995*a*). *Structure*, **3**, 535–540. 10.1016/s0969-2126(01)00187-3 8590014

[bb17] Kleywegt, G. J. & Jones, T. A. (1995*b*). *Proceedings of the CCP4 Study Weekend. Making the Most of Your Model*, edited by W. N. Hunter, J. M. Thornton & S. Bailey, pp. 11–24. Warrington: Daresbury Laboratory.

[bb18] Knight, S., Andersson, I. & Brändén, C. I. (1990). *Proceedings of the CCP4 Study Weekend. Accuracy and Reliability of Macromolecular Crystal Structures*, edited by K. Henrick, D. S. Moss & I. J. Tickle, pp. 83–90. Warrington: Daresbury Laboratory.

[bb19] Korkhov, V. M. & Tate, C. G. (2009). *Acta Cryst.* D**65**, 186–192. 10.1107/S0907444908036640 PMC2631640 19171974

[bb20] Miller, C. (2007). *Science*, **315**, 459. 10.1126/science.315.5811.459b 17255494

[bb21] Schreuder, H. A., Curmi, P. M. G., Cascio, D. & Eisenberg, D. (1990). *Proceedings of the CCP4 Study Weekend. Accuracy and Reliability of Macromolecular Crystal Structures*, edited by K. Henrick, D. S. Moss & I. J. Tickle, pp. 73–82. Warrington: Daresbury Laboratory.

[bb22] Smith, J. L. *et al.* (2009). In preparation.

